# Experience-dependent critical period for synaptic plasticity in the rat olfactory amygdala

**DOI:** 10.3389/fnsyn.2026.1779075

**Published:** 2026-05-15

**Authors:** Belén Aburto-Ponce, Pedro Fernández-Aburto, Jorge Mpodozis, Juan Bacigalupo, Magdalena Sanhueza

**Affiliations:** Departamento de Biología, Facultad de Ciencias, Universidad de Chile, Santiago, Chile

**Keywords:** smell, olfactory amygdala, synaptic plasticity, postnatal sensory maturation, sensitive period

## Abstract

**Introduction:**

The amygdala is involved in processing and memory of emotional stimuli. The cortical regions of the amygdala are situated medially to the piriform cortex (PC) and are also considered part of the olfactory system, which may explain why scents can evoke strong emotional responses and trigger vivid memories. Similarly to the PC, the anterior cortical nucleus of the amygdala (ACo) is a three-layered structure receiving direct input from the main olfactory bulb (MOB) through the lateral olfactory tract (LOT), without a thalamic relay. While activity processing, plasticity and behavioral relevance of the PC have been extensively studied, evidence on the functional properties of the olfactory amygdala is scarce. ACo participates in the innate response to aversive stimuli and could be involved in olfactory emotional learning. Primary cortices of different sensory modalities display early periods in which afferent connections are particularly plastic, and this is crucial for network maturation. Here, we studied the plastic properties of LOT-ACo synapses during olfactory system maturation, by assessing long-term potentiation (LTP) induction during the first weeks of life.

**Methods:**

We performed field recordings of LOT-ACo synaptic potentials in brain slices from rats of 6 to 35 postnatal days (P6-P35) to assess LTP induction by theta-burst stimulation. To investigate if the developmental regulation of plasticity can be altered by early sensory deprivation, we performed unilateral naris occlusion.

**Results:**

We found that LTP was significantly higher between P16-P25 (reaching ~30%) compared to earlier or later stages. Interestingly, LTP was missing around the end of the first postnatal month (P26-35). Furthermore, we demonstrated that LTP induction at one month of age was recovered in both hemispheres in rats that were subjected to unilateral sensory deprivation.

**Discussion:**

This result suggests the existence of a critical period for LTP induction in olfactory connections to the amygdala. Therefore, ACo may not only be involved in innate responses to biologically relevant odorants, but could also participate in early experience-dependent associative learning. Moreover, olfactory deprivation could extend the critical period for plasticity, making the system prone to undergo plastic changes at later developmental stages.

## Introduction

Olfaction is essential for the survival of animals, providing important cues about their chemical surroundings. Furthermore, olfactory perception is deeply intertwined with emotions, as scents can evoke strong feelings and memories, influencing behavior and decisions (Herz, 2009). Olfactory processing starts at the olfactory epithelium with the binding of volatile odorants to receptors in the cilia of olfactory sensory neurons. Each sensory neuron expresses one type of odorant receptor (Buck and Axel, 1991). Neurons having the same receptor class are distributed along the whole epithelium and their axons converge into 1–2 glomeruli of the main olfactory bulb (MOB; Mombaerts et al., 1996), thereby creating a map where odors are encoded in a combinatorial way (Malnic et al., 1999; Menini et al., 2004). Mitral and Tufted cells from the MOB send their axons through the lateral olfactory tract (LOT) to several cortical regions, among them, the piriform cortex (PC), the largest and more studied olfactory cortex, and the amygdala.

**Figure 1:**
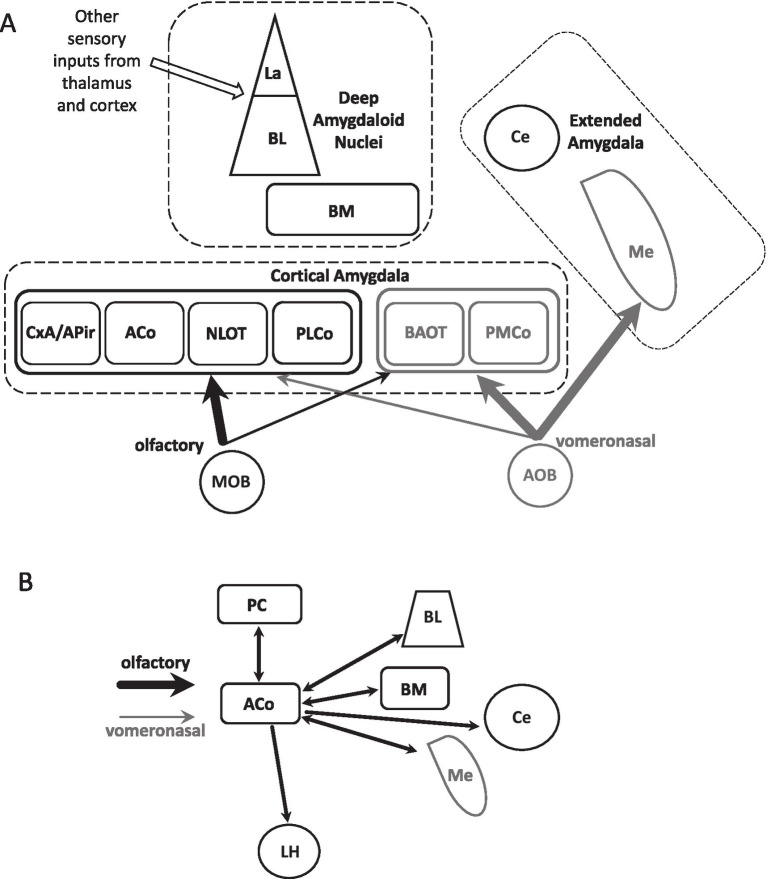
Main components of the amygdala complex and ACo intra/extra amygdala connectivity. **(A)** Schematics of the three main divisions of the amygdala complex and their principal constituents. The deep nuclei, usually grouped under the term “basolateral amygdala division”, include the lateral (La), basolateral (BL), and basomedial (BM) nuclei. These nuclei receive thalamic and cortical inputs of different modalities, except olfaction. Connections from the main olfactory bulb (MOB; black arrows) arrive at cortical amygdala regions, including the anterior and posterolateral cortical nuclei (ACo, PLCo), the cortico-amygdala transition zone (CxA), the amygdalo-piriform transition area (APir), and the nucleus of the lateral olfactory tract (NLOT). Meanwhile, vomeronasal inputs (grey arrows) from the accessory olfactory bulb (AOB) mostly target the cortical bed nucleus of the accessory olfactory tract (BAOT) and the posteromedial cortical nucleus (PMCo). Thick and thin arrows indicate massive and sparse afferent connectivity, respectively. The central (Ce) and medial (Me) nuclei are part of the “extended amygdala” and constitute two major output structures of the amygdala complex. The deep nuclei and those of the extended amygdala have several subdivisions not shown in the illustration. **(B)** Diagram of some of ACo main input and output pathways, including both intra- and extra-amygdala connections. For simplicity, the mostly reciprocal connections with other cortical regions of the olfactory amygdala are not included. PC, piriform cortex; LH, lateral hypothalamus.

The amygdala complex is a heterogenous group of nuclei and cortical structures receiving inputs from different sensory modalities (Figure 1A; Swanson and Petrovich, 1998; McDonald, 1998; Pitkänen et al., 2000; Olucha-Bordonau et al., 2015). Following the definitions of Olucha-Bordonau et al. (2015) the basolateral division of the amygdala (BLA) is comprised by the lateral (La), basal (BL) and basomedial (BM, also known as accessory basal) nuclei, which may display anterior/posterior, medial/lateral and dorsal/ventral subdivisions. We are explicit with this choice as differences in the definition of the basolateral complex and their constituents among authors may generate some confusion. BLA is a target of several sensory cortices and thalamic nuclei, visual, somatosensory, auditory and gustatory, and is involved in forms of emotional learning as fear conditioning (LeDoux, 2000, 2007; Sah et al., 2003; Olucha-Bordonau et al., 2015; Duvarci and Pare, 2014). In contrast to other sensory modalities, olfactory inputs reach the amygdala directly at its cortical regions, without a thalamic relay (Price, 1973; McDonald, 1998). Two main LOT targets at the amygdala are the anterior (ACo) and posterolateral (PLCo) cortical nuclei (Olucha-Bordonau et al., 2015), denoted by other authors as COa and COp (Pitkänen et al., 2000).

As illustrated in Figure 1A, the amygdala complex also includes cortical and nuclear structures receiving direct vomeronasal inputs originated in the accessory olfactory bulb (AOB). This apparent segregation between the olfactory (black arrows) and vomeronasal circuits (gray arrows) is not absolute, since in addition to the main afferents, each region receives a small contribution from the complementary chemosensory system (thick and thin arrows). Finally, the central (Ce) and medial (Me) nuclei are part of the “extended amygdala” and constitute two major output regions connecting to the hypothalamus and brain stem structures involved in the generation of autonomic and behavioral responses to biologically relevant stimuli. Moreover, Me is considered part of the vomeronasal system as it receives strong inputs from the AOB. Figure 1B summarizes the major ACo input and output connections, excluding the strong intracortical amygdala connectivity.

From a developmental point of view, the cortical and deep nuclei derive from the ventral pallium, instead PC originates from the lateral pallium, and the extended amygdala has a subpallial origin, closer to the striatum (Olucha-Bordonau et al., 2015).

Although the properties and involvement of the BLA in processing and memory of biologically relevant sensory inputs have been studied for decades, functional data from the cortical amygdala and its involvement in biologically relevant olfactory processing is still scarce. While the PC primarily encodes odor discrimination and learned associations (Poo and Isaacson, 2009), olfactory connections to the amygdala may be involved in processing stimuli with biological relevance, innate valence and behavioral salience (Cadiz-Moretti et al., 2017; Root et al., 2014). These two streams use overlapping MOB output neurons but are differentially organized, weighted and functionally specialized.

In contrast to the spatially organized sensory projections to the MOB, in which different odors activate characteristic subsets of glomeruli, olfactory connections to the PC are diffuse and broadly distributed (Ghosh et al., 2011; Sosulski et al., 2011). This organization and the strong recurrent associational connectivity allow the PC to operate as a combinatorial array for olfactory discrimination and memory (Stettler and Axel, 2009; Poo and Isaacson, 2009; Wilson and Sullivan, 2011; Franks et al., 2011). On the other hand, available information on olfactory processing in the cortical nuclei of the amygdala and their behavioral relevance is limited. Pioneering work showed that while PC receives connections from the whole MOB, afferent inputs to the olfactory amygdala are mainly (but not exclusively) from dorsal glomeruli (Miyamichi et al., 2011; but see Root et al., 2014) and their ablation preferentially disrupts innate responses to aversive volatile odorants (Kobayakawa el al., 2007). This suggested that the cortical amygdala is involved in the generation of innate odor-driven behaviors.

In contrast to the diffuse organization of MOB projections to PC, olfactory inputs to PLCo are arranged in stereotypic, partially overlapping patches, suggesting the preservation of a topographic sensorial map (Sosulski et al., 2011; Ghosh et al., 2011) hardwired during development and resembling the connectivity in primary cortices of other sensory modalities.

Later, Root et al. (2014) showed that ACo neurons are in fact activated by aversive odors, and their optical reactivation is sufficient to generate the appropriate behavioral response. For their part, PLCo neurons were involved in the response to appetitive odorants. These observations reinforced the idea that the cortical amygdala is critical for generating innate odor-driven behaviors, but do not preclude its participation in the processing of neutral odorants and learned associations. Interestingly, recent work using retrograde and anterograde tracing demonstrated that ACo receives connections originating from mitral cells along the whole MOB (Cadiz-Moretti et al., 2017), suggesting that ACo neurons may be responsive to many odorants and not only to those with innate valence. In line with this result, a predator odor or a novel neutral scent induced comparable c-Fos expression in ACo (Dielenberg et al., 2001).

Finally, electrophysiological recordings in behaving rats indicated that ACo may also participate in olfactory fear conditioning (Sevelinges et al., 2004), pointing to ACo as a possible locus of olfactory-related emotional learning.

To the best of our knowledge, the only available evidence on the local circuit functional properties of the olfactory amygdala came from our electrophysiological studies in slices (Sanhueza and Bacigalupo, 2005; Vera et al., 2014). There, we reported that the majority of ACo layer II neurons display intrinsic subthreshold membrane potential oscillations and resonance in the theta frequency range, which are commonly observed phenomena in learning and memory-related structures as the hippocampus, the entorhinal cortex and the basolateral amygdala.

Long-term potentiation (LTP) is a form of synaptic plasticity underlying memory (Poo et al., 2016). It consists of a sustained enhancement of synaptic transmission that can be induced by high frequency presynaptic stimulation (Bliss and Collingridge, 1993). In the PC, as well as in primary cortices of other sensory modalities, there is a limited time window during postnatal development that is characterized by a highly dynamic synaptic plasticity which in some cases is restricted to this period, whereas in other cases it can continue to occur, albeit at a lower rate. This time span constitutes a critical period for LTP induction (Franks and Isaacson, 2005; Poo and Isaacson, 2009; Hensch, 2005; Crair and Malenka, 1995; Kirkwood et al., 1995; Barkat et al., 2011) that parallels the specific timing of sensory maturation shaped by the animal’s experiences.

Rat pups are strongly dependent on olfaction during early post-natal life. It has been determined that the critical period in the PC declines by the first postnatal month (Poo and Isaacson, 2009; Franks and Isaacson, 2005), but studies on olfactory amygdala are lacking. Here, we used brain slices from rats of different ages to investigate LTP induction in the afferent connections to ACo during the first weeks of life. We found that plasticity induction indeed occurs, although it disappears at around a month of age, indicating the existence of a critical period in the olfactory amygdala. To assess whether the establishment of this plasticity window depends on early olfactory experience, we conducted sensory deprivation by unilateral naris occlusion. We found that the ability to generate LTP was preserved after 1 month, suggesting that the critical period was extended. Interestingly, plasticity recovery was observed in both hemispheres.

## Methods

### Ethical approval

Animal care and experimental procedures were approved by the Bio-Ethical Committee of the Faculty of Sciences, University of Chile, according to the ethical rules of the Biosafety Policy Manual of the National Fund for Scientific and Technological Development (FONDECYT).

### Preparation of slices

We used 6–34 days old Sprague–Dawley male rats. They were anesthetized by isoflurane inhalation and sacrificed by decapitation. The brain was quickly removed and transferred to a dissection chamber containing (mM): 83 NaCl, 2.5 KCl, 3.3 MgSO_4_, 0.5 CaCl_2_, 25 NaHCO_3_, 1.25 NaH_2_PO_4_, 10 glucose, 72 sucrose; pH 7.4 (by saturation with 95% O_2_ and 5% CO_2_ at 0–4 °C). Coronal sections (400 μm) containing ACo were cut with a Vibratome (Pelco). The slices were maintained at 36 °C in oxygenated artificial cerebrospinal fluid (ACSF) for at least 2 h before starting the experiments. ACSF contained (mM): 125 NaCl, 2.5 KCl, 1 MgCl_2_, 2 CaCl_2_, 25 NaHCO_3_, 1.25 NaH_2_PO_4_ and 10 glucose; pH 7.4.

### Electrophysiology

Slices were immersed in a recording chamber containing ~2 mL ACSF. A total volume of 15 mL of ACSF (bubbled with 95% O_2_, 5% CO_2_, at 34 °C) was recirculated during each experiment using a peristaltic pump. Ag/AgCl recording electrodes in borosilicate capillaries (0.3–0.4 MΩ) filled with ACSF were positioned on the outermost region of layer I (layer Ia) of ACo. Afferent fibers were stimulated by current pulses (Isostim stimulator A320, WPI), using two bipolar electrodes (Platinum/Iridium, 0.2–0.3 MΩ) positioned in the same layer, at each side of the recording electrode (~200 μm from it). Field recordings of excitatory postsynaptic potentials (fEPSPs) were obtained with a differential amplifier (1800 A-M SYSTEMS) and filtered to a bandwidth of 1 Hz–5 kHz. Except for input–output experiments, stimulus intensity was set to evoke ~60% of the maximum response or the appearance of a population spike. Basal stimulation frequency was 0.05 Hz. To ensure that only glutamate transmission was being assessed, the GABA-A receptor antagonist picrotoxin (100 μM) was added to the recirculating external solution.

LTP was induced in one synaptic pathway by 4 trains of theta-burst stimulation (TBS), separated by 30 s. Each TBS train consisted of 10 bursts of stimuli, separated by 200 ms (5 Hz), and each burst comprised 5 pulses at 100 Hz. The stimulus intensity used to induce LTP was the same as for basal transmission recording. The second synaptic pathway was used as a control for recordings stability. The data was acquired using the Igor Pro 6.12 (Wavemetrics) software.

### Sensory deprivation

Olfactory deprivation was conducted on neonatal rats (P1–P4). Unilateral nostril occlusion was performed under anesthesia by hypothermia (Phifer and Terry, 1986) using an adhesive plug or cauterization. Following occlusion, animals recovered in a warmed incubator (30–33 °C) before being returned to the dam until weaning (P20). Animals were subsequently housed with littermates until experimental use.

### Quantification of data and statistical methods

Synaptic transmission was evaluated by measuring the slope of fEPSP, using Igor Pro 6.12. As in certain experiments some rundown of synaptic strength was detected in the control pathway, the percentage of LTP (% LTP) in each experiment was quantified with respect to control pathway in the interval between 20 and 30 min after TBS. Experiments with more than 10% rundown were discarded. To determine whether a significant LTP was induced in an experiment, test and control responses (normalized to their respective baseline values; 10 min before TBS) were compared in the same interval, using a Student’s *t* test for paired data.

To compare % LTP between different age ranges, a one-way ANOVA and Bonferroni *post hoc* test was used. The average values of normalized synaptic transmission in test and control pathways as well as the percentage of change by age range are expressed as mean ± standard error of the mean (SEM). The criterion for statistical significance was *p* < 0.05. The statistical analysis was performed with Graphpad Prism 5.01 software.

### Histology

The lateral olfactory tract was labeled *in vitro* using a modified protocol from Chang et al. (2000). P11-P13 and P20 rats were anesthetized by isoflurane and rapidly decapitated. The brain was removed and transferred to a chamber containing ice-cold dissection solution (bubbled with 95% O_2_/5% CO_2_). A portion of the brain containing ACo was transferred to a maintenance chamber at 25 °C, filled with (in mM): 124 NaCl, 5 KCl, 2 MgCl_2_, 10 glucose, 1.25 NaH_2_PO_4_, 2 CaCl_2_, 26 NaHCO_3_, and left to recover for at least 30 min. The tissue was then placed in a Petri dish containing ACSF, with its anterior side facing upwards, and a biocytin crystal (Ne-biotinyl-L-lysine, Sigma) was placed on the LOT of one hemisphere, while the other hemisphere was used as a control. Tissue was then returned to the maintenance chamber and left there for 6–8 h. Afterwards, it was fixed for 2 days in 4% paraformaldehyde in 0.1 M PBS, pH 7.4. Finally, it was embedded in 0.1 M PBS with 30% sucrose and cut in a coronal plane (60 μm slices) with a microtome (Leitz).

For biocytin detection, sections were placed in 6-well plates and processed with the avidin-biotin-peroxidase complex method. They were washed for 10 min in PBS and subsequently incubated for 20 min in 30% hydrogen peroxide to saturate the endogenous peroxidase activity. After washing 3 times for 10 min, slices were incubated with avidin-biocytin in PBS with 0.3% Triton X-100 for 3 h and then washed with PBS (3 × 10 min). They were then left to react with diaminobenzidine (DAB) 0.4% in PBS, in the presence of 0.01% hydrogen peroxide and 0.04% nickel chloride, for 5–10 min and washed with PBS (3 × 15 min) to stop the reaction. Finally, the sections were mounted on microscope slides, dried for 1 day and covered with coverslips (slices not stained with Nissl) or prepared for Nissl staining.

For Nissl staining, rats of P11 and P20 days were anesthetized with ketamine /xylaxine 4 mg /0.2 mg and 4 mg /0.8 mg per 100 g, respectively. They were perfused with 4% PFA in PBS. The brain was extracted and embedded in 30% sucrose in PBS to be later sectioned into 60 μm slices using a Leitz microtome. Both the sections obtained this way as those previously revealed with DAB, were mounted on gelatinized slides and dried for 1 day. After this, they were left in chloroform for 1 h, hydrated through a series of graded alcohols (100, 95, 70 and 50% for 3, 3, 4 and 4 min, respectively) in distilled water for 4 min and immediately stained with cresyl violet (0.75%). Sections were then washed with distilled water and dehydrated with graded alcohols (50, 70, 95 and 100%, x 2; 1 min each). Finally, they were allowed to stand in Xylol for 1 h, covered with a coverslip and Permount (Sigma-Aldrich). The images of these samples were obtained with a confocal microscope.

## Results

To assess whether there is a critical period for LTP induction in the connections from the MOB to ACo, we conducted field electrophysiological recordings in rat brain slices. Before performing the functional study, it was necessary to have an orientation of the position of ACo and the location of the more external region of layer I (layer Ia), where the LOT axons run (Price, 1973). For this, we used histological methods to stain ACo neurons and LOT fibers in brain sections and identified the cortical amygdala in coronal slices by using a rat brain atlas and other published descriptions (Paxinos and Watson, 1986; Olucha-Bordonau et al., 2015).

### ACo structure and innervation by LOT axons

**Figure 2:**
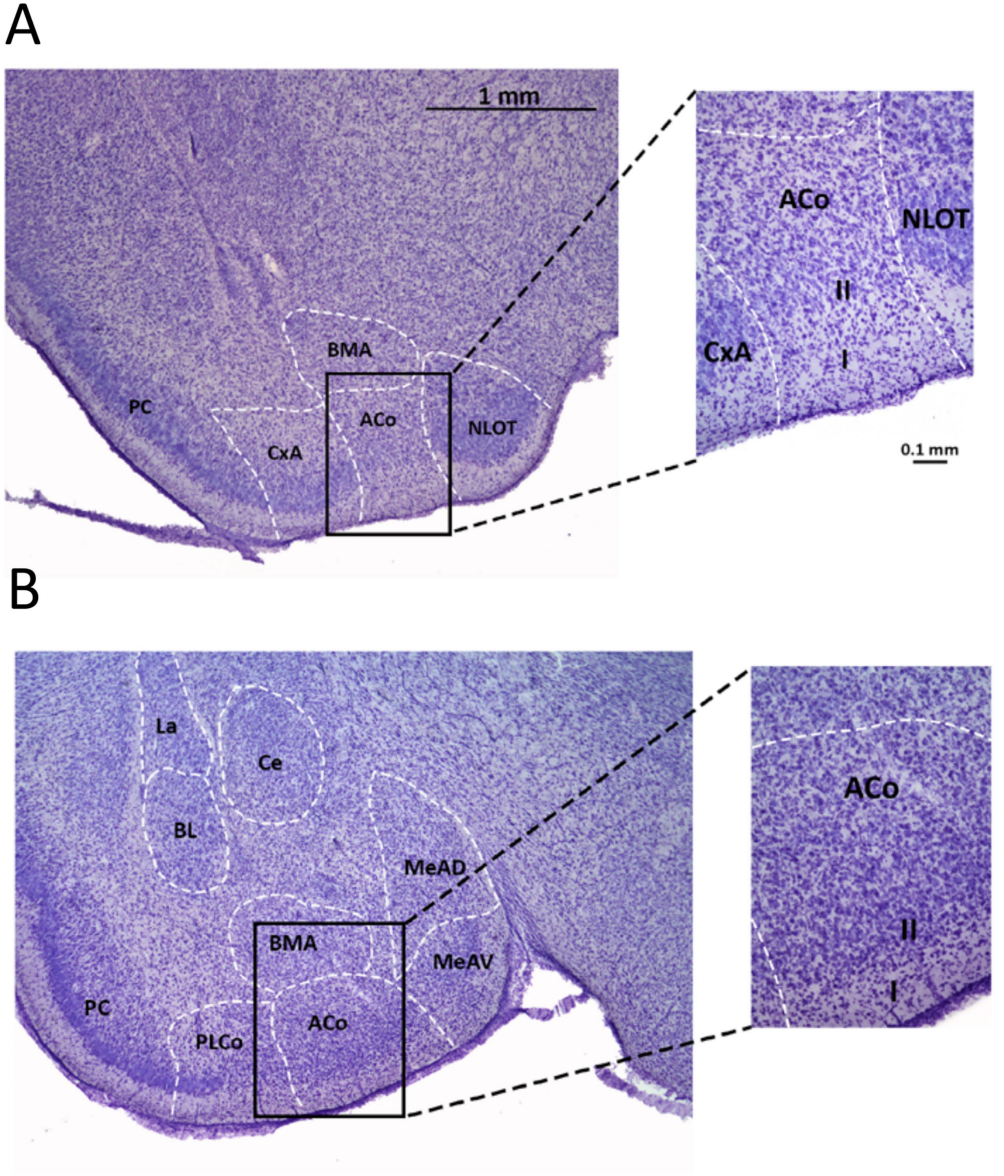
Location and layers of the olfactory cortical amygdala. Nissl staining of two brain coronal sections (80 μm width; P11) at different anteroposterior locations, containing the PC and regions of the cortical amygdala. In both slices, layer I (low density zone) can be distinguished from layer II (higher density of cell bodies). **(A)** The image shows the amygdala anterior cortical nucleus, ACo; surrounded by the cortex-amygdala transition zone (CxA) and the bed nucleus of the accessory olfactory tract (BAOT), according to Olucha-Bordonau et al. (2015), Paxinos and Watson (1986). Immediately dorsal to ACo lies the anterior part of the basomedial nucleus (BMA). Bregma −1.44. Inset: close-up of the sector framed with the rectangle in **A**, indicating the location of layers I and II. **(B)** Slice posterior to A; ACo is flanked by the posterolateral cortical nucleus (PLCo) and the anteroventral subdivision of the medial nucleus (MeAV). La and BL, lateral and basolateral nuclei. Ce, central nucleus. MeAD, anterodorsal part of the medial nucleus. Bregma −2.16. Inset: enlargement of the region framed by the rectangle in **B**.

To infer the size and location of layers I and II in neonatal/weaning rats, we Nissl-stained brain slices from two different ages, P11 and P20. We chose the midpoint of the two earliest periods at which LTP would be assessed, to test whether the cortical amygdala structures, their layers and MOB projections at these stages show similar anatomical properties as those described for adult rats (Price, 1973; Paxinos and Watson, 1986; Olucha-Bordonau et al., 2015), and to ensure that the stimulation and recording electrodes will be positioned correctly. Figure 2 shows Nissl staining in coronal slices from a P11 animal. In Figures 2A,B, it is possible to identify the olfactory cortical regions at two different anteroposterior locations by the spatial distribution of their respective Nissl-stained cells. In the piriform cortex (PC), the cells are densely packed in layer II and are mostly absent in layer I. Two layers can also be distinguished in ACo by their different cell densities, lower in layer I than in layer II. In ACo, layer II cells are more dispersed than in PC. This characteristic spatial distribution of the cells in ACo is the reason why it is classified as a “cortex-like” structure (Sah et al., 2003).

**Figure 3:**
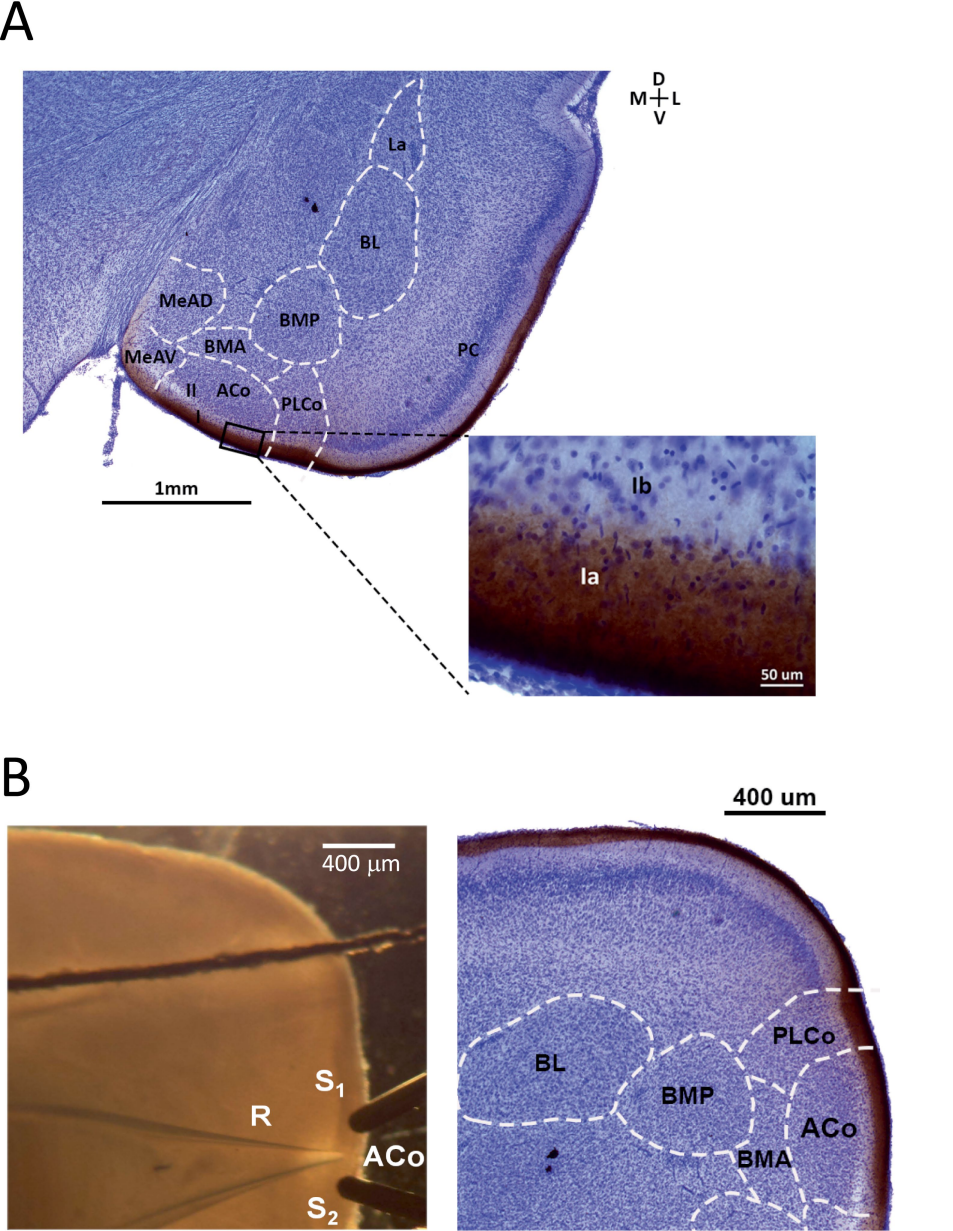
Location and layers of ACo and experimental setup. **(A)** Nissl stain of a coronal brain slice (80 μm width) containing ACo and PC, obtained from a P20 rat. Layer Ia containing the LOT axons was stained by a crystal of biotin revealed with DAB. Inset shows a detail of the region delimited by the rectangle, corresponding to the most superficial sublayers of ACo. BMA/P, anterior/posterior division of the basomedial group. Bregma −2.52. **(B)** Left, experimental arrangement displaying the position of the stimulation (S_1_, S_2_) and recording (R) electrodes in layer Ia. Right, brain section of a comparable anteroposterior position obtained from an animal of similar age, displaying biotin stain of LOT axons and Nissl counterstain.

As Nissl only stains the cell bodies, we examined the position of LOT axons by labeling the fibers in rat brains *in vitro* by means of a crystal of the anterograde biotin tracer revealed with DAB (Chang et al., 2000). The position of these axons in the outermost region of layer I can be clearly identified (Figure 3A), both in the PC and the cortical amygdala, resembling observations in adult animals (Price, 1973). These results allowed us to recognize the LOT fibers in ACo layer Ia under a stereomicroscope in the electrophysiological experiments.

### Critical period for synaptic plasticity in ACo during the first month of age

After determining the approximate location of LOT axons in ACo, we explored whether there is a temporal window after birth in which afferent synaptic transmission is especially susceptible to undergo potentiation. We examined LTP induction by electrical stimulation of the LOT axons in coronal brain slices during the first month of life. Figure 3B illustrates the experimental arrangement, including a Nissl-stained section from a similar position and with biotin-labelled LOT fibers, for comparison. Two stimulation electrodes (S1, S2) were positioned in layer Ia of ACo, at both sides of a recording electrode (R); this layer is clearly distinguishable in the slices under a stereomicroscope. To ensure that the evaluated plasticity corresponded to glutamatergic and not to GABAergic innervations, we performed our measurements in the presence of the GABA-A receptor antagonist picrotoxin (100 μM).

**Figure 4:**
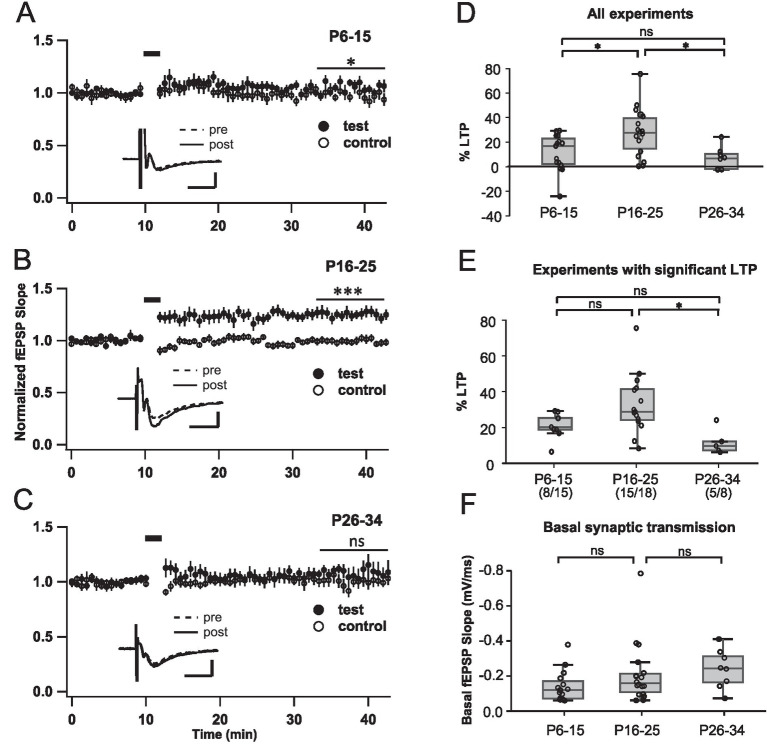
The degree of potentiation in the cortical amygdala depends on animal age. **(A–C)** Summary plots displaying the effect of TBS (horizontal bars) at different postnatal periods. fEPSP slope was normalized with respect to basal values (10 min before stimulation). Test and control pathway responses were compared along the last 10 min of recordings (20–30 min after TBS; horizontal lines). Insets in **(A–C)**: superimposed average basal (0–10 min) and potentiated responses (20–30 min after TBS), for sample experiments. Calibration: 0.2 mV, 5 ms. *N* = 15 slices (11 rats), for A; *N* = 18 slices (13 rats), for **B**; *N* = 8 slices (6 rats), for **C**. **(D)** Percent LTP calculated with respect to the control pathway, comprising those in which a significant LTP was induced as well as those in which it was not. To determine if LTP was induced in an experiment, responses evoked in the test and control pathways during the last 10 min were compared (30 records per pathway). **(E)** Percent LTP, considering only experiments in which a significant increase was observed. **(F)** Basal synaptic strength (10 min before stimulation), for the same experiments in **A–C**. ^*^p < 0.5; ^***^p < 0.0001.

We chose to examine LTP induction over three time periods: 6–15, 16–25, 26–34 days (a detailed breakdown of all experiments performed between P6 and P34 is provided in Supplementary Figure 1). Figures 4A–C presents the summary plots obtained for rat slices of each period, which display an evident age-dependent behavior. To determine if, on average, the LTP protocol caused a statistically significant potentiation, we compared the normalized test and control responses along the last 10 min of recordings (20–30 min after TBS) for each experiment and compared the paired data. We detected a small, though significant fEPSP increment in the youngest group (test, 1.07 ± 0.03; control, 0.97 ± 0.04; *N* = 15; Student’s *t* test for paired data, *p* = 0.03), and a more pronounced increase in the following set of animals (test, 1.26 ± 0.05; control, 1.00 ± 0.02; *N* = 18, *p* < 0.0001). However, change was detected between the test and control pathway for the older group (test, 1.07 ± 0.05; control, 1.00 ± 0.05, *N* = 8, *p* = 0.07), indicating that, on average, LTP was missing at this age.

We then quantified the percentage of LTP (% LTP) induced at the different stages (Figure 4D). This plot includes both experiments in which LTP was induced (statistically significant increase in transmission with respect to control pathway) and those where the TBS produced no significant potentiation. The animals of the intermediate group (P16-25) presented the greatest increase (27.7 ± 4.5%), significantly different than that of the early (P6-15) and late (P25-34) groups, with changes of 11.3 ± 3.8% (*p* = 0.019) and 6.6 ± 3.2% (*p* = 0.012), respectively (one-way ANOVA, Bonferroni *post-hoc* test). The early and late groups, on the other hand, showed no difference between themselves. This result reveals the existence of a critical period in which LTP in the synapses of LOT axons to ACo is maximal, and after which LTP drastically declines.

The larger average potentiation in P16-25 could be due to a higher probability of induction, to an increase in experiments where LTP was induced, or both. To evaluate this, we quantified the % LTP considering only experiments where the TBS stimulation induced significant potentiation (Figure 4E). Significant differences were found between the groups (*p* = 0.009, one-way ANOVA); in the first two groups, LTP was not statistically different (P6-P15: 21.1 ± 2.4% and P16-25: 32.9 ± 4.3%; *p* = 0.125, Bonferroni post-hoc test), while the third group (the oldest) showed significantly lower mean potentiation (11.9 ± 3.3%) compared to the P26-35 animals (*p* = 0.013). The first and third groups were similar (*p* = 0.653). In addition, the likelihood of inducing LTP in individual experiments was higher during the P16-25 period compared to previous and later ages (Figure 4E; 53% for P6-P15, 83% for P16-P25 and 63% for P26-P25).

To test if the difference in LTP could be related to age-dependent variations in basal synaptic transmission, we measured the slope of the postsynaptic potentials evoked in each experiment by stimuli producing 60% of the maximum response, corresponding to the condition in which TBS was applied. We found no significant differences in basal transmission between the three groups (Figure 4F; P6-15: −0.14 ± 0.02 mV/ms, P16-25: −0.20 ± 0.035 mV/ms and P26-34, −0.23 ± 0.05 mV/ms, one-way ANOVA, *p* = 0.2), contrasting with the induction of LTP. In fact, we observed a trend towards an increase in transmission with age, which would be expected to facilitate instead of hindering LTP. Therefore, the lack of LTP at later stages cannot be explained by differences in basal transmission but is a manifestation of the loss of plastic properties of the afferent synaptic inputs to the ACo.

Our results indicate that there is an interval in the postnatal development of infant-juvenile animals (between P16 and P25) in which both the likelihood of inducing LTP and their magnitude is larger than for earlier and later ages. Interestingly, the midpoint of this critical period of higher plasticity approximately coincides with weaning (around P21; Nashizumi, 2025), a hallmark in rat development where they start eating solid food and exploring their surroundings.

Notably, these results resemble observations of the evolution of plastic properties in the afferent connections to PC, where LTP also disappears by the end of the first month of life (Poo and Isaacson, 2009). Thus, our observations suggest that, in addition to its participation in processing innately aversive odorants, ACo may be involved in age-dependent associative learning.

### The critical period in ACo is extended after unilateral olfactory deprivation

**Figure 5:**
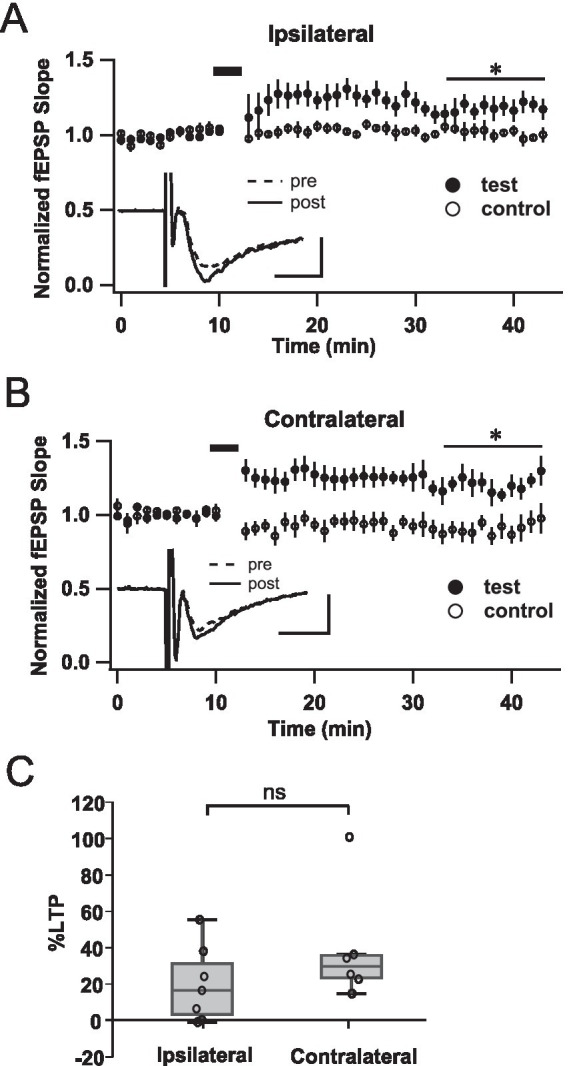
LTP at later stages is recovered in both hemispheres after early unilateral naris occlusion. **(A, B)** Summary plots displaying the effect of TBS in slices from P25-35 animals, for the ipsilateral and contralateral hemispheres to occlusion, respectively. Test and control pathway responses were compared over the last 10 min of recordings (20–30 min after TBS; horizontal lines). *N* = 7 slices (5 rats), for **A**; *N* = 6 slices (6 rats), for **B**. **(C)** Percent LTP calculated with respect to the control pathway (20–30 min after TBS), including all experiments.

In different sensory systems, including olfaction, the critical period depends on postnatal experience (Nashizumi, 2025; Mallick et al., 2024; Chung et al., 2017; Kirkwood et al., 1996). In the olfactory system, sensory neurons extend their axons exclusively to the ipsilateral MOB, thus unilateral naris occlusion has been extensively used as a model for olfactory deprivation, with the “spared” hemisphere considered as an internal control (Brunjes, 1994; Coppola, 2012; Nashizumi, 2025). Following this procedure, Franks and Isaacson (2005) showed that deprivation facilitates LTP induction during the critical period in the ipsilateral (deprived) PC compared to the contralateral (spared) hemisphere. Therefore, we conducted unilateral naris occlusion in neonatal rats and studied LTP induction in both hemispheres at P25-35 (Figures 5A,B), to assess a possible modification of the critical period in the ipsilateral ACo.

As previously, to determine if the TBS protocol caused a statistically significant potentiation in the ipsi- and contralateral hemispheres at this stage, we compared synaptic responses in test and control pathways along the last 10 min of recordings (20–30 min after TBS). In contrast to naïve rats that on average did not display potentiation at this age, we detected a significant increment in fEPSP in the ipsilateral cortex (control, 1.03 ± 0.02; test, 1.23 ± 0.07; *N* = 8; Student’s *t* test for paired data, *p* = 0.039). Surprisingly, in the contralateral hemisphere transmission enhancement was even more pronounced (control, 0.92 ± 0.05; test, 1.26 ± 0.07; *N* = 18, *p* = 0.015).

The summary graph in Figure 5C shows a detailed breakdown of the % LTP obtained in all experiments performed on hemispheres ipsilateral or contralateral to the occluded nasal cavity. The average potentiation was 19.9 ± 7.9% and 39.0 ± 12.8%, in the ipsilateral and contralateral hemispheres respectively, displaying no statistically significant differences (*p* = 0.217; One-way ANOVA).

Therefore, the plastic properties of the LOT-ACo synapses that were normally lost around 1 month of age were recovered bilaterally, with significant LTP induced in both hemispheres.

Finally, we evaluated basal synaptic strength in both hemispheres by comparing the input–output curves (stimulation intensity—fEPSP slope) and found no significant difference between hemispheres (see Supplementary Figure 2). Moreover, the baseline transmission in the test pathway before applying TBS in the specific experiments of Figure 5C, was not significantly different (Ipsilateral: −0.24 ± 0.05 mV/ms; contralateral: −0.17 ± 0.03 mV/ms; *p* = 0.22, *t*-student test). Finally, when compared with naïve animals of similar age, no differences were found between groups either (one-way ANOVA, *p* = 0.403), indicating that the absence of LTP in non-deprived animals was not related to basal synaptic strength.

Overall, our results indicate that the duration of the critical period in the olfactory amygdala can be bilaterally extended by unilateral sensory deprivation, suggesting different roles of PC and ACo in olfactory processing and memory.

## Discussion

In the present study, we provide electrophysiological evidence that synaptic plasticity at main olfactory bulb (MOB) afferent synapses onto neurons of the anterior cortical nucleus of the amygdala (ACo) is subject to marked developmental regulation. We identify a postnatal time window during which LTP at LOT–ACo synapses is most robust, followed by a pronounced decline toward the end of the first postnatal month. Moreover, we show that early olfactory deprivation by unilateral naris occlusion extends this window of plasticity, restoring the capacity for LTP induction at ages at which normally it no longer occurs. Together, these results indicate that synaptic plasticity in the olfactory amygdala is both developmentally constrained and experience-dependent, supporting the existence of a critical period in this limbic olfactory structure.

### A developmental critical period for synaptic plasticity in the anterior cortical amygdala

Our data demonstrate that LTP at LOT–ACo synapses is maximal between postnatal days 16 and 25, whereas potentiation is weaker at earlier ages and decreases sharply after approximately postnatal day 26. This loss of plasticity cannot be attributed to changes in basal synaptic transmission. Although basal synaptic strength did not differ significantly across age groups, it displayed a clear developmental trend toward increased excitatory transmission. From a mechanistic perspective, stronger synaptic transmission would be expected to facilitate LTP induction by promoting postsynaptic depolarization and calcium influx during high-frequency stimulation, a key requirement for LTP induction (Bliss and Collingridge, 1993). Contrary to this expectation, LTP declined markedly toward the end of the first postnatal month despite preserved or increasing basal transmission. This dissociation suggests that the loss of plasticity with age in ACo reflects a developmental change in the intrinsic capacity of glutamatergic synapses to undergo long-term modification, rather than alterations in baseline synaptic efficacy. On the other hand, the weaker LTP in the youngest group (from postnatal 6 to 15) could be in part due to insufficient innervation by LOT axons.

Critical periods for synaptic plasticity are a hallmark of sensory system development and have been extensively characterized in primary sensory cortices. In the olfactory system, a comparable developmental decline in LTP has been reported in the piriform cortex (PC), where afferent synaptic plasticity decreases toward the end of the first postnatal month (Franks and Isaacson, 2005; Poo and Isaacson, 2009). Our findings extend this principle to the olfactory amygdala and indicate that ACo follows a similar developmental trajectory, despite its distinct embryonic origin, location and presumed functional role.

Although both structures receive direct input from the MOB and display a laminar, cortex-like organization in the adult brain, PC derives from the lateral pallium, whereas ACo is part of the amygdala complex and originates primarily from the ventral pallium (Olucha-Bordonau et al., 2015; Medina and Abellán, 2009). Thus, similarities in sensory afferents set do not necessarily imply shared developmental programs or plasticity mechanisms. From this perspective, the presence of a temporally restricted window for LTP induction in ACo is particularly noteworthy, as it indicates that critical-period–like regulation of synaptic plasticity can emerge in a structure traditionally associated with emotional processing. Notably, the period of highest plasticity coincides with weaning, a developmental stage characterized by increased exploratory behavior and autonomy (Nashizumi, 2025).

### Mechanisms of LTP and critical period regulation in ACo

As mentioned, physiological data on cortical amygdala is scarce. Most evidence comes from behavioral studies, including circuit tracing, innate odor valence coding, behavioral modulation and activity-induced gene expression. To our knowledge, this is the first report on LTP induced by electrical stimulation in the cortical regions of the amygdala, and in the present work we have focused on the developmental regulation of this form of plasticity, specifically in the ACo. While further studies are necessary to assess the underlying cellular mechanisms of this form of LTP and the physiological bases of the critical period, we may hypothesize about possible shared pathways with related sensory cortices like the PC, as well as other, more studied regions of the amygdala complex as the basolateral division (BLA).

We used a theta-burst stimulation (TBS) protocol that mimics the network rhythm observed in rat olfactory structures during exploratory sniffing, and which is synchronized with theta activity in memory-related networks such as the hippocampus and the amygdala (reviewed in Tort et al., 2018). TBS-induced NMDA receptor (NMDAR)-dependent LTP has been demonstrated in several brain cortical and subcortical regions, with the CA3-CA1 hippocampal synapses representing the most studied model (Nicoll, 2017). In BLA, LTP is strongly NMDA-dependent and closely tied to fear learning (Chapman et al., 2003; Rodrigues et al., 2004a). In thalamic inputs to BLA, however, LTP also relies on a contribution of L-type voltage-dependent channels. The cellular signaling pathways involved in NMDAR-dependent LTP in BLA and related emotional memories involve calcium/calmodulin-dependent kinase II (CaMKII), protein kinase A (PKA), gene expression, protein synthesis, extracellular signaling-related kinases (ERKs), cyclic AMP-response element binding protein (CREB) and brain derived neurotrophic factor (BDNF), among others (Rodrigues et al., 2004b; Ehrlich and Josselyn, 2016).

NMDAR-dependent LTP is also induced in PC by TBS applied to afferent or associative inputs (Kanter and Haberly, 1990; Franks and Isaacson, 2005). While the mechanism of LTP in PC has not been studied in as much detail as in the hippocampus or BLA, olfactory cortex pyramidal neurons share canonical cortical glutamatergic signaling pathways and a similar cascade can be invoked when interpreting piriform plasticity. Moreover, forms of olfactory learning have been shown to involve some critical components such as CaMKII and BDNF (Ghosh et al., 2016; Jones et al., 2007). Therefore, it is reasonable to hypothesize that LTP in LOT-ACo synapses might rely on similar mechanisms.

Although the biological mechanisms underlying a critical period for LTP in ACo remain unknown, several developmental processes provide a plausible framework. In PC, experience-dependent postnatal maturation and critical period is accompanied by a progressive increase in AMPA receptor contribution relative to NMDA receptor-mediated transmission (Franks and Isaacson, 2005). As described in PC and other cortical sensory systems, developmental changes in NMDA receptor signaling—including a decrease in the relative proportion of GluN2B versus GluN2A subunit composition and associated Ca^2+^ dynamics—may progressively constrain synaptic potentiation (Quinlan et al., 1999; Philpot et al., 2001; Paoletti et al., 2013; Franks and Isaacson, 2005). Moreover, regulation of NMDAR composition induced by olfactory learning limits subsequent synaptic plasticity (Quinlan et al., 2004).

A second likely contributor is the maturation of GABAergic inhibition, which is closely linked to critical-period timing across brain regions (Hensch, 2005) and undergoes significant postnatal refinement in the amygdala, including changes in GABA-A receptor function and inhibitory synaptic dynamics (Ehrlich et al., 2013; Bessières et al., 2019). In our experiments, GABA-A receptor-mediated transmission was pharmacologically blocked, indicating that the observed critical period for LTP reflects properties intrinsic to excitatory synapses rather than acute inhibitory control. Similar experimental conditions were used in studies of PC, where a developmental decline in LTP was also observed despite blockade of GABAergic transmission (Franks and Isaacson, 2005; Poo and Isaacson, 2009). Moreover, recent work indicates that maturation of inhibitory circuits in PC proceeds relatively slowly and is not strongly affected by unilateral naris occlusion (Pardo et al., 2018). These findings contrast with the visual system, where the onset and closure of critical periods are tightly linked to the maturation of GABAergic inhibition (Hensch, 2005; Jiang et al., 2005; Levelt and Hübener, 2012). Together, our results suggest that in olfactory cortical circuits, including ACo, critical period regulation may rely more strongly on developmental changes in glutamatergic synapses or intracellular signaling pathways, rather than on inhibitory circuit maturation per se. However, our results do not exclude the contribution of inhibition to the regulation of the critical period during network development.

BDNF signaling is one of the central molecular mechanisms implicated in critical period timing and closure across several brain systems (Park and Poo, 2013). BDNF regulates the maturation of GABAergic transmission and operates as a driver of neural plasticity in glutamatergic synapses, promoting activity-dependent dendritic spine stabilization and reduction of structural plasticity, thus contributing both to the opening and closure of plasticity windows (Huang et al., 1999; Kaneko et al., 2012). Homeostatic plasticity and metaplasticity mechanisms may further shape this process, by regulating LTP threshold (Lee and Kirkwood, 2019; Yu et al., 2019; Guo and Lau, 2022), particularly in olfactory circuits, where sensory deprivation induces compensatory synaptic adjustments that preserve baseline transmission while altering plasticity capacity (Franks and Isaacson, 2005). Finally, progressive structural stabilization -potentially mediated by extracellular matrix components and perineuronal nets-, may impose physical and molecular constraints on synaptic remodeling, contributing to the closure of heightened plasticity with age (Gogolla et al., 2009; Sorg et al., 2016).

### Experience-dependent regulation and extension of the critical period

A defining feature of critical periods is their sensitivity to sensory experience (Nashizumi, 2025). In line with this principle, we found that early unilateral naris occlusion preserved the capacity for LTP induction in ACo beyond the age at which plasticity is normally lost. Importantly, when basal synaptic transmission was examined specifically in the set of experiments in which LTP was assessed, no significant differences were detected between naïve animals and deprived animals, nor between hemispheres. Thus, the recovery of LTP following deprivation cannot be attributed to changes in basal synaptic efficacy.

Moreover, the preservation of basal transmission suggests that the deprivation procedure did not cause profound or irreversible disruptions of LOT–ACo connectivity (Coppola, 2012). Instead, compensatory or homeostatic mechanisms may act to stabilize synaptic strength in response to reduced afferent activity during development. Under these conditions, deprivation appears to selectively maintain or reinstate the mechanisms that permit synaptic modification, rather than altering baseline transmission.

### Bilateral effects of unilateral olfactory deprivation

Unilateral naris occlusion has been widely used as a model of olfactory deprivation based on the largely ipsilateral projection of olfactory sensory neurons to the MOB, with the contralateral hemisphere commonly treated as an internal control. While this assumption is valid at the level of primary afferents, our data indicate that it does not fully apply to downstream limbic structures such as the amygdala.

We observed a bilateral recovery of LTP in deprived animals, with particularly robust potentiation in the hemisphere contralateral to the occluded naris. This finding suggests that interhemispheric interactions, intra-amygdaloid connectivity, or indirect coupling through hypothalamic and cortical targets, may allow unilateral sensory manipulations to influence both hemispheres (Cadiz-Moretti et al., 2017; Olucha-Bordonau et al., 2015). Such bilateral effects highlight important differences between PC and cortical amygdala circuitry and underscore the need for caution when using the “spared hemisphere” as a control in deprivation paradigms targeting olfactory regions.

### Comparison with experience-dependent plasticity in the piriform cortex

A comparison with deprivation studies in the PC reveals both shared principles and important distinctions. One similarity is that both in the present study and in Franks and Isaacson (2005) basal synaptic transmission is comparable between hemispheres after early unilateral naris occlusion. On the other hand, they observed that LTP induction by a weak TBS protocol was facilitated in the hemisphere ipsilateral to the occluded naris compared to the contralateral hemisphere. This hemispheric difference was not observed when stronger stimulation protocols were used, comparable to the protocol employed in the present study. Based on these findings, Franks and Isaacson proposed that early deprivation lowers the threshold for LTP induction rather than enhancing the expression of LTP itself. Importantly, their experiments were conducted during the critical period, whereas the present study demonstrates recovery of robust LTP after the critical period has normally closed. Furthermore, Franks and Isaacson reported that at later stages (P26–35), weak TBS could still induce LTP in the contralateral hemisphere, albeit to a lesser extent than during peak plasticity. Together with subsequent work showing that LTP is largely absent around the end of the first postnatal month in naïve animals (Poo and Isaacson, 2009), these findings suggest that early deprivation may extend the critical period for synaptic plasticity in PC, at least in the contralateral hemisphere. In the present study, LTP recovery in both ACo hemispheres reached levels comparable to those observed during the period of maximal plasticity, highlighting potential differences in circuit organization and plasticity regulation between PC and ACo. However, the different conditions and protocols in both studies make a direct comparison difficult.

### Embryonic origin and neurodevelopmental properties of cortical and deep amygdala nuclei

The developmental interpretation of synaptic plasticity in ACo can be further refined by considering its embryonic origin. Although the cortical amygdala shares structural and functional features with the lateropallial piriform cortex, these regions are developmentally distinct and differentially connected. The ventropallial origin of the cortical amygdala places it at the interface between cortical and subcortical systems, likely exposing it to distinct developmental programs governing circuit assembly, inhibitory maturation, and neuromodulatory regulation. The cortical amygdala may follow a different trajectory shaped by the integration of olfactory inputs with emotional and motivational circuits. Under this framework, early postnatal plasticity in the ACo may reflect not only intrinsic synaptic permissiveness but also an ongoing process of circuit integration specific to ventropallial derivatives.

Postnatal neurogenesis has been documented in the amygdala, particularly in the BLA complex and adjacent regions, where neurons continue to be generated and integrated during early postnatal life and even in adults (Bernier et al., 2002; Jhaveri et al., 2018). During the first postnatal month, BLA principal neurons display dramatic changes in both electrophysiological properties and morphology, including a two-fold increase in somatic size and a progressive increment in spine density from P7 to P28, the age at which adult levels are reached (Ryan et al., 2016). In contrast, there is currently no region-specific characterization of neuronal birth, maturation, or integration in the cortical amygdala. Nevertheless, given its cortical-like organization, it is likely that principal neurons are generated prenatally, with postnatal development dominated by synaptogenesis, interneuron maturation, and sensory-driven circuit refinement, as described in related olfactory cortical areas. Under this framework, early postnatal stages may reflect a period in which cortical amygdala neurons are not yet fully functionally stabilized but instead participate in an actively assembling network. Indeed, PC neurons reach already at P15 the maximum spine density in distal regions receiving inputs from LOT, while in the basal and proximal apical dendrites innervated by associative connections, the density continues to increase beyond the first month of life. Therefore, in ACo, associative connectivity may still be in the process of maturation during the first postnatal weeks. This could confer a heightened capacity for synaptic potentiation, which is consistent with a developmental window of enhanced plasticity. Conversely, the subsequent reduction in LTP may reflect progressive circuit stabilization and maturation. Therefore, ACo may partially resemble PC, but follow a different developmental logic in terms of its connections with other ventropallial regions such as BLA. The absence of direct developmental data for this cortical region highlights a significant gap in our understanding of olfactory amygdala circuit formation.

### Functional specificity of ACo and its implications for emotional learning

ACo occupies a strategic position within olfactory–limbic networks. It receives a strong input from the MOB and sends projections to several key regions involved in emotional and motivational processing, including the basolateral division, the central and medial nuclei, the lateral hypothalamus, and the bed nucleus of the stria terminalis (Cadiz-Moretti et al., 2017; Olucha-Bordonau et al., 2015). These output pathways place ACo in a position to influence the generation of emotional and autonomic responses to biologically relevant olfactory stimuli, whether innate or acquired.

Although ACo has been associated with innate odor-driven behaviors, several lines of evidence support a broader functional role. *In vivo* electrophysiological recordings have shown that after olfactory fear conditioning, synaptic potentials evoked by LOT stimulation are persistently potentiated in ACo and BLA (Sevelinges et al., 2004). Our findings provide a developmental and synaptic framework for these observations, suggesting that ACo circuits may undergo experience-dependent plasticity during a defined postnatal window. Together, these results support the idea that ACo may contribute not only to innate responses but also to the formation of odor–emotion associations, particularly during early life.

While direct functional data on the role of cortical amygdala in learning is scarce, a link with processing and memory of safety signals involving olfactory cues is supported by gene activity mapping, as the expression of plasticity-related genes after behavioral training involving defensive/aggressive behaviors (Knapska et al., 2007). However, more detailed information that distinguishes between the olfactory and vomeronasal cortical regions is needed. Interestingly, by analyzing c-Fos activity, Keller et al. (2004) proposed that specifically CoA and the medial amygdala (Me) participate in olfactory social recognition memory in lambs.

This raises the question of how ACo participates in the generation of biologically relevant olfactory memories supported by engrams involving the rest of the amygdala. As for other sensory modalities, BLA activation is critical for olfactory fear conditioning (Cousens and Otto, 1998; Rosenkranz and Grace, 2002; Walker et al., 2005). BLA complex (specifically, La and BL) and Ce receive nociceptive inputs related to the unconditioned stimulus (foot-shock) (Knapska et al., 2007; Olucha-Bordonau et al., 2015) and the simultaneous activation of ACo by the conditioned stimulus (odor) is expected to activate the ACo intra-amygdala, mostly reciprocal connectivity (Figure 1), which may allow activity integration and plasticity.

During the first month of life, odor-guided attraction and aversion learning are developmentally regulated in rats. Pups are primed to generate attachment to the caregiver, displaying a reduced ability to acquire odor aversion. To study the underlying circuits and developmental regulation of this behavior, Sullivan and coworkers (Sullivan, 2001; Moriceau and Sullivan, 2004; Moriceau and Sullivan, 2005; Moriceau et al., 2006) used a mammalian imprinting model, where a neutral odor was paired with an aversive stimulus (electric shock). They showed that until approximately day P11, the period in which the neonates remain confined to the nest, this association does not produce fear conditioning, but rather a paradoxical approach response to the odorant. During this “sensitive period,” the immaturity of stress pathways favors learning preference over aversion. This paradoxical conditioning is independent of the amygdala complex but relies on the noradrenergic innervation of the MOB and anterior piriform cortex by the locus coeruleus (Moriceau and Sullivan, 2004). During the transition period to weaning (around P12-15), when the animals begin to temporarily leave the nest (pre-weanling period), the same protocol can produce either approach or aversion depending on whether the mother is present at the time of conditioning (Moriceau and Sullivan, 2006). In this period, the caregiver’s presence prevents the increase in corticosterone. After weaning, around P21-23, fear conditioning always occurs. Moreover, in both the pre-weanling and post-weanling periods the capability to generate odor-related fear memories correlates with the activation of ACo and BLA, as well as of Me. Interestingly, electrical stimulation of ACo outputs to BLA fails to induce synaptic plasticity in this region in slices obtained during the sensitive period. In contrast, in slices from 11–18 days old rats, an age at which it becomes possible to generate aversive memories, stimulation of this pathway can evoke plasticity in slices (Thompson et al., 2008).

Overall, evidence suggests that, in this paradigm, amygdala complex activation drives the developmental switch from attraction to aversion and supports a critical role of ACo-mediated stimulation of the deep nuclei in odor–valence learning. Remarkably, the age at which fear conditioning begins to be reliably induced, coincides with the period in which maximal LTP is observed in LOT-ACo connections.

In addition to ACo drive, maturation of stress-related pathways, including glucocorticoid signaling via the hypothalamic–pituitary–adrenal axis, are necessary for reaching high enough activity levels in BLA. Glucocorticoids and neuromodulators such as dopamine are known to increase neuronal excitability in BLA by regulating local GABAergic circuits (Ehrlich et al., 2009) and thus facilitate synaptic plasticity and the formation of emotional memory (Duvarci and Paré, 2007; Rodrigues et al., 2009).

Our results raise the question of whether plasticity in LOT-ACo synapses would continue to contribute to odor-related memories after critical period closure, as suggested by electrophysiological recordings following fear-conditioning in adult rats (Sevelinges et al., 2004). It is possible to propose that, as for the PC (Kanter and Haberly, 1993), the recruitment of the mature intracortical associative circuit by strong afferent stimulation could exert a cooperative effect in LTP induction. Moreover, as ACo also receives direct inputs from the thalamic nuclei associated with nociception (Olucha-Bordonau et al., 2015), it could be hypothesized that the coincidence of afferent olfactory activity and nociceptive inputs may cause associative plasticity in ACo principal cells, which could assist with fear memory formation.

Interestingly, ACo might participate not only in fear-related memories. A recent *in vivo* study demonstrated the involvement of ACo neurons in odor-guided reward-directed learning (Shiotani et al., 2026). Using a go/no-go test, this work showed that a population of ACo neurons display sustained responses selectively to the rewarded odor, predicting animal behavior and, in some cases, remaining active during reward.

A possible pathway underlying the participation of ACo in odor-reward associations may be its strong and reciprocal connections with the adjacent basomedial nucleus (BM). BM neurons differentiate safe and aggressive contexts, and as targets of medial prefrontal cortex, take part in the top-down control of anxiety and cued-conditioned fear (Adhikari et al., 2015). Moreover, a direct contribution of ACo in this modulation cannot be discarded. An interesting possibility would be that the concurrent activation of ACo neurons by inputs from the ventral tegmental area (VTA) (Majkutewicz et al., 2010) and the excitatory projection from ACo to the nucleus accumbens (Cadiz-Moretti et al., 2017), might interact with the dopamine mesolimbic reward system. Finally, ACo connections with the lateral hypothalamus (LH) could also contribute to reward-related responses (Stuber and Wise, 2016). While LH is associated with the execution of fear responses through escape/avoidance behaviors, it could also influence preference by regulating feeding, arousal, and motivation, through intermingled neuronal populations, among which orexin/hypocretin neurons may be central (Tyree and de Lecea, 2017).

Overall, the evidence suggests that odor-dependent CoA activation and its functional connectivity with other amygdala nuclei, and perhaps extra-amygdala structures, are involved in the formation and stabilization of engrams that encode odor valency learning. The establishment of conditioned aversion during the first postnatal month coincides with the period of maximal plasticity in afferent connections to ACo. Additional experiments are needed to assess if after closure of the critical period, ACo may preserve plastic properties if assisted by the concurrent recruitment of associative circuits and intra-amygdala recurrent connections.

A possible interpretation of the present findings is that early olfactory experience may produce memory traces that are durable in affective significance but comparatively limited in contextual specificity. ACo is well positioned to support such a process, as an olfactory-recipient amygdala region likely involved in assigning emotional salience to odor cues. Within this framework, heightened developmental plasticity in afferent connections to ACo could favor the long-term encoding of biologically relevant odor experience, whereas the relative immaturity of piriform associative circuitry and hippocampal–cortical memory systems early in life may constrain the precision with which those experiences are bound to context and later retrieved (Franks et al., 2011; Travaglia et al., 2016; Alberini and Travaglia, 2017). Odors are especially effective in evoking vivid and emotionally laden autobiographical memories (Larsson and Willander, 2009), consistent with the so-called “Proust effect,” a phenomenon mainly described in humans. Moreover, odor-evoked memories are often highly emotional and appear to be biased toward earlier life periods, suggesting that early olfactory experience may leave particularly enduring traces (Larsson and Willander, 2009). This interpretation remains speculative, but it is consistent with the idea that early learning can persist as latent traces even when not retained in a fully explicit or richly contextualized form (Travaglia et al., 2016).

Finally, projections from ACo to the medial amygdala provide a potential anatomical substrate for the integration of olfactory and vomeronasal activity. Recent studies have shown that these connections may support associative interactions between volatile odor cues processed by the main olfactory system and social or pheromonal signals conveyed by the vomeronasal system (Keshavarzi et al., 2015; Guthman and Vera, 2016). Although the present study focuses on MOB inputs, such convergence may further expand the functional relevance of experience-dependent plasticity in ACo, particularly in the context of emotionally salient multisensory associations.

### Limitations and broader implications of olfactory deprivation

Finally, it is important to consider that unilateral naris occlusion may induce changes beyond reduced sensory input. Previous studies have reported structural alterations in the MOB, LOT and downstream connectivity following early deprivation (Coppola, 2012). While the preservation of basal synaptic transmission in deprived animals suggests that major connectivity disruptions are unlikely, such structural changes may nonetheless contribute to circuit-level reorganization. The coexistence of preserved basal transmission and enhanced plasticity suggests that deprivation induces a network state that favors synaptic modification without compromising overall synaptic integrity. Future studies combining anatomical, physiological, and molecular approaches will be required to disentangle these contributions.

## Data Availability

The raw data supporting the conclusions of this article will be made available by the authors, without undue reservation.
