# Supplementary material for: Experience-dependent critical period for synaptic plasticity in the rat olfactory amygdala
Source: Front Synaptic Neurosci. 2026 May 15;18:1779075. doi: 10.3389/fnsyn.2026.1779075 (PMC13219369; doi:10.3389/fnsyn.2026.1779075)
Supplement: Supplementary file 1 [file Supplementary_File_1.docx]

**Supplementary Material**


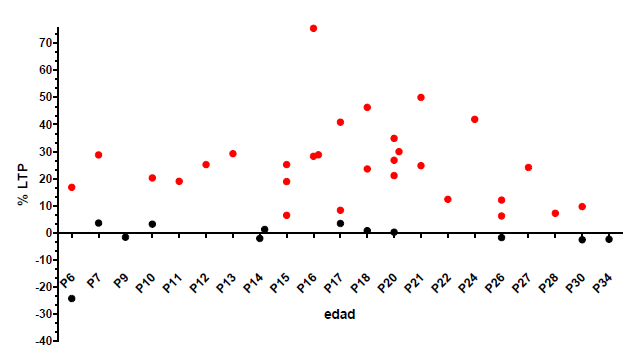


**Supplementary Figure 1.**

Detailed breakdown of TBS-induced potentiation in each experiment performed between P6 and P34 (n = 41). The percentage of LTP was calculated relative to the control pathway (20–30 min post-TBS). The graph includes both experiments in which a statistically significant increase was observed (red dots) and those in which it was not (black dots).

**Supplementary Figure 2. Basal synaptic strength in LOT-ACo connections at P21-25 is similar in both hemispheres after early unilateral olfactory deprivation.**

Average input-output curves: fEPSP slope (mV/ms) as a function of stimulation intensity for slices from hemispheres ipsilateral (continuous line, N = 5) and contralateral (dotted line, N = 5) to the occluded nostril. No statistically significant difference was observed between the slope and Y-intercept of the curves (p = 0.7 and p = 0.4, respectively; linear regression and ANCOVA).
